# Transmission is a Noticeable Cause of Resistance Among Treated Tuberculosis Patients in Shanghai, China

**DOI:** 10.1038/s41598-017-08061-3

**Published:** 2017-08-09

**Authors:** Chijioke A. Nsofor, Qi Jiang, Jie Wu, Mingyu Gan, Qingyun Liu, Tianyu Zuo, Guofeng Zhu, Qian Gao

**Affiliations:** 1 School of Basic Medicine, Key Laboratory of Medical Molecular Virology of Ministries of Education and Health, Fudan University, Shanghai, China; 2 Department of Tuberculosis Control, Shanghai Municipal Center for Disease Control and Prevention, Shanghai, China

## Abstract

It is generally believed that drug resistance among treated tuberculosis (TB) patients is as a result of acquired drug resistance due to inappropriate treatment. Previous studies have shown that primary drug resistance caused by transmission also plays a role among treated cases. Differentiating the two types of drug resistance will help in developing appropriate strategies for control of drug resistant tuberculosis. In this study, we tested the hypothesis that drug resistance among treated TB patients is mainly caused by primary resistance rather than acquired resistance. Defining resistance profiles by molecular drug susceptibility test, we used Unit Variable Number Tandem Repeats (VNTR) to genotype and Whole Genome Sequencing (WGS) to confirm the accordance of the first and last *Mycobacterium tuberculosis* isolates from treated pulmonary TB patients in Shanghai from 2009–2015. Among 81 patients with increasing drug resistance, out of 390 patients enrolled, paired isolates from 59.3% (48/81) had different VNTR patterns indicating primary drug resistance. Our results have demonstrated that primary resistance due to exogenous reinfection is the major cause of drug resistance among treated TB patients in Shanghai; thus, strategies aimed at preventing and interrupting transmission are urgently needed to effectively reduce the epidemic of drug resistant tuberculosis.

## Introduction

China is among the 22 high tuberculosis (TB) and multidrug resistant tuberculosis (MDR-TB) burden countries in the world^1, 2^. World Health Organization (WHO) recently estimated about 480 000 new cases of multidrug-resistant TB (MDR-TB), and reported that India, China and the Russian Federation accounted for 45% of the combined total of 580 000 MDR cases^2^. Numerous reports from different regions across China in recent years showed MDR-TB rates ranged from 4.0% to 16.6%^3–7^. And the nationwide drug resistance survey in 2007 showed that the proportion of MDR-TB in smear-positive pulmonary TB patients was 5.71% among those were new cases^2^.

Drug resistance in *Mycobacterium tuberculosis (M. tuberculosis)* may result from two mechanisms: inadequate therapy that enables selection of drug-resistant strains (acquired resistance) or infection with a drug resistant *M. tuberculosis* strain (primary resistance)^8^. Acquired resistance is presumed to be responsible for the majority of cases of drug resistance among treated TB patients^9–11^; however, this may be a misleading assumption, particularly as studies have shown that exogenous reinfection by transmitting drug resistance strains and mixed infection are associated with changing *M. tuberculosis* drug-resistance patterns and thus, may be a significant factor in development of drug resistance among treated cases^12–14^. Distinguishing between acquired and primary resistance is important for creating a strategy to address the expanding drug resistant TB epidemic. If the majority of cases of drug resistance are as a result of acquired resistance, emphasis must be placed on strengthening effective treatment. If, however, primary resistance is a major factor, the creation and implementation of transmission control programs must be added as a critical component of the control strategy.

This study was therefore designed to determine whether the major drug resistant cases among treated TB patients is actually caused by acquired resistance or due to primary drug resistance. We genotyped paired clinical isolates of *M. tuberculosis* from treated individuals with pulmonary tuberculosis in Shanghai, China. We utilized both genotyping method (variable number tandem repeats, VNTR) and high-throughput genomic epidemiological tool -whole genome sequencing (WGS) to determine the rates of primary and acquired resistance among treated patients.

## Results

### Study Population

**Figure 1:**
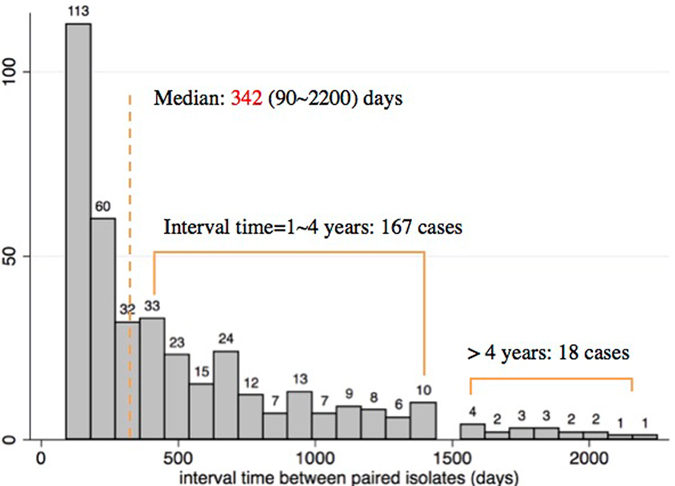
The time difference of sample collection between paired isolates. We enrolled the first isolate and the last isolate from the same patients and interval of sputum collection less than 90 days were excluded. The median interval was 342 days, ranging from 90 to 2200 days.

Within the period of 2009 to 2015, about 10,514 pulmonary TB patients were diagnosed and treated in the 31 designated district tuberculosis hospitals in Shanghai. For patients with more than two isolates collected, we selected the first and the last isolate in analysis. Isolates with DNA of poor quality failing molecular drug susceptibility test (DST) or VNTR genotyping were excluded. In all, 780 *M. tuberculosis* isolates from 390 patients were analyzed in this study. The age of the patients ranged from 15–89 years with average age of 34 years, and males accounted for 80.2% of the patients. The time of collecting sputum specimens ranges from 90–2200 days with average being 342 days (Fig. 1). When the first samples were collected before treatment, 21% (83/390) patients had TB treatment history and were registered as retreated patients. Compared to all the culture-positive patients, enrolled cases had more retreated cases (21% vs 9%, p < 0.001) and MDR patients (23% vs 7%, p < 0.001); but no difference was observed in age and gender between these two groups.

### Drug Resistance Pattern Changes of Treated TB patients

**Figure 2:**
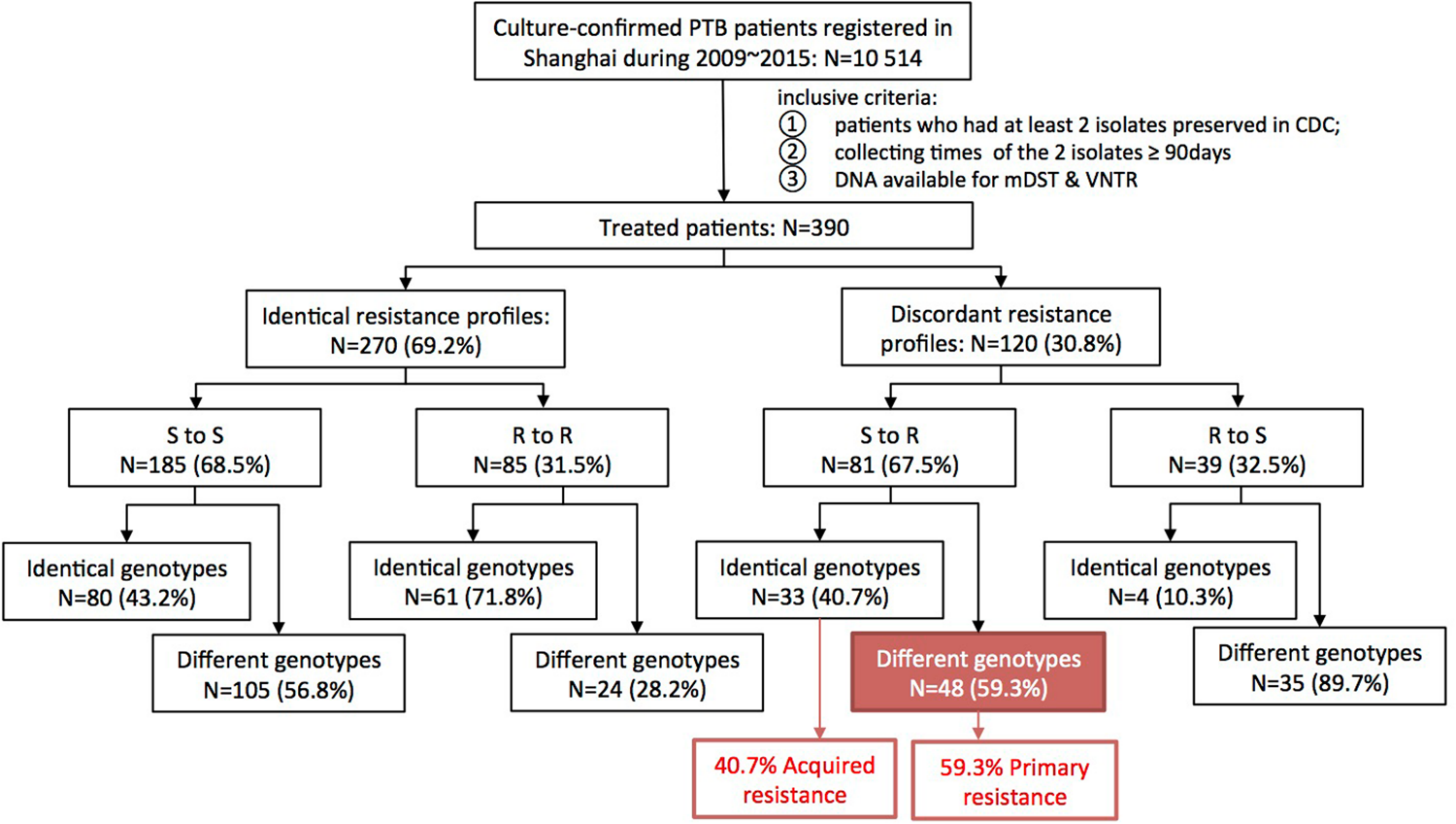
The Work Flow of the Study. Treated patients with at least two strains stored were enrolled; resistance changes were defined based on genotypes; different VNTR genotypes of the same patient indicate reinfection while identical genotypes of paired isolates suggested acquired resistance.

Since the molecular DST is more rapid and can detect resistant mutations in both first and second line drugs, resistance changes were based on molecular DST. Table 1 shows the summary of the phenotypic and molecular DST of the isolates against Isoniazid and Rifampicin. We compared molecular DST results of the first isolate with the second isolate from each patient. The initial isolates from 232 patients were pan-sensitive while those from 90 patients were MDR-TB. Most treated patients (69.2%, 270/390) had paired isolates showing the same molecular DST results, and were grouped as patients with identical resistance, in which 185 patients were pan-sensitive and 85 resistant. Patients whose first and subsequent isolates showed discordant molecular DST results were classified as patients with changing resistance, accounting for 30.8% (120/390) of treated patients. Among them, isolates from 81 (67.5%) patients changed from sensitive to resistant (S-R) while isolates from 39 (32.5%) patients changed from resistant to sensitive (R-S). (Fig. 2).

**Table 1:** The summary of the Phenotypic DST and Molecular DST of the isolates.

Phenotype Genotype	Resistance		Sensitive		Total	SE	SP
	MT	WT	MT	WT			
RIF	200	9	54	517	780	0.957	0.905
INH	234	45	8	493	780	0.839	0.984

KEY: RIF = Rifampicin, INH = Isoniazid, MT = Mutant type, WT = Wild type, SE = Sensitivity, SP = Specificity.

### Primary Resistance is the Major Cause of Increasing Drug Resistance

To investigate for drug resistance due to primary or acquired resistance, we genotyped the isolates using VNTR (9 + 3) loci. The results showed that, 59.3% (48/81) of paired isolates from the S-R group had different VNTR pattern in first and second isolates (Fig. 2). Among these 48 patients, isolates from more than half (25/48, 52.1%) had the different VNTR in four or more VNTR loci, while eight pairs had difference in one VNTR hypervariable locus and five pairs differed at one regular locus. Also, nine and six pairs differed at two and three VNTR loci respectively. Therefore, based on these VNTR differences, we concluded that these 59.3% (48/81) patients had primary drug resistance.

**Figure 3:**
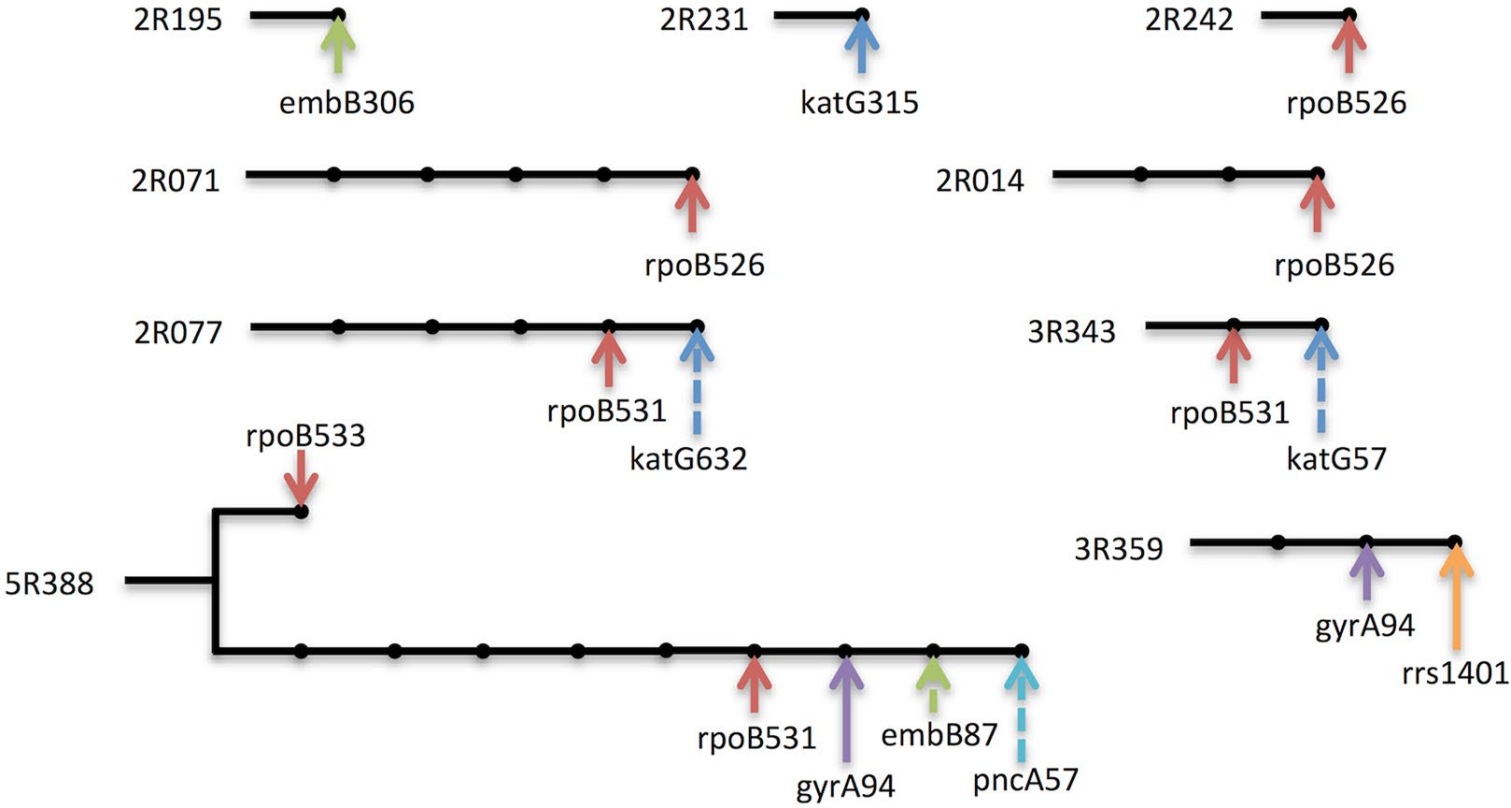
Accumulation of acquired resistance mutations as shown by whole genome sequencing. Lines or branches following the patient ID showed the distances of paired isolated. Each dot represented one SNP and arrows with different colors indicated mutations in different resistance genes. Solid arrows were detected and dash arrows were missed mutations by molecular DST.

Among the 81 patients with increasing drug resistance (S-R), acquired drug resistance was observed in 40.7% (33/81) i.e. their paired isolates had identical VNTR pattern in first and second isolate including nine cases changed from pan-sensitive to mono-resistant, two cases from mono-resistant to MDR, and five cases from MDR to extensively drug-resistant (XDR) (Fig. 2). To further investigate the genomic similarities and the accumulation of the acquired resistance mutations of this isolates, the whole genome sequencing was conducted. The result of 11 (33.3%) out of 33 pairs of isolates with acquired resistance mutations showed that the paired isolates were actually identical with single nucleotide polymorphisms (SNPs) differing from zero to five. The most common acquired mutations were *rpoB*526 & *rpoB*531 while about 25% (4/16) of new acquired mutations were missed by molecular DST (Fig. 3A).

### High Reinfection Rates in patients with identical resistance or R-S group

To further show that exogenous reinfection is a major contributing factor in the transmission of tuberculosis, we also genotyped the paired isolates from the patients with identical resistance. We observed 56.8% (105/185) reinfection rate among patients of this group, showing a similar rate of 59.3% observed in 81 patients with increasing drug resistance (Fig. 2). Also, 89.7% (35/39) reinfection rate was observed among the 39 patients in the R-S group suggesting a relatively high rate of reinfection in our study setting due to transmission, however, isolates from four patients in this group had identical VNTR pattern, one of them were found to be parallel evolution with different mutations in *katG* (Fig. 3). Furthermore, among the patients within the S-R group, we investigated further to ascertain the rate of reinfection or mixed infection among the subpopulation. We found that isolates from 7.4% (6/81) of these patients had multiple VNTR bands in their first isolate indicating mixed infection.

## Discussion

In this study, we evaluated the cause of drug resistance among treated tuberculosis patients in Shanghai, China. Our result showed that based on VNTR(9 + 3) typing, 59.3% treated patients with increasing drug resistance had primary drug resistance as a result of exogenous reinfection while 40.7% were due to acquired resistance. Also we observed 56.8% reinfection among patients whose paired *M. tuberculosis* isolates were drug pan-sensitive.

Contrary to the prevailing dogma that major drug resistant tuberculosis among treated patients is caused by acquired resistance presumably because the infecting strain had developed resistance due to inappropriate treatment^9–11, 15, 16^; we have shown that a greater number of treated patients (59.3%) in our study setting had resistant TB due to transmission of drug resistant *M. tuberculosis* strains. Transmission and re-infection have been shown to be driving the TB epidemic^17, 18^. WHO drug resistance surveillance data was confirmed that as many or more MDR-TB cases now occur among previously untreated patients clearly indicating a serious result from transmission^19^. Furthermore, previous data from different parts of China and other parts of the world also highlighted the role of transmission in disseminating drug resistant tuberculosis^2, 3, 12, 17, 18, 20–22^. For example, in a large-scale population-based study conducted across five provinces of China, Yang *et al*.^17^ concluded that a considerable proportion of tuberculosis cases in China, including MDR tuberculosis cases, were due to recent transmission of *M. tuberculosis*. Similarly, in another retrospective study recently conducted in Shanghai, Yang *et al*.^18^ observed that 73% of all MDR tuberculosis cases in Shanghai were due to transmission.

One major difference between our study and others is the approach we used; unlike the previous authors, we genotyped paired isolates from each patient and thus were able to distinguish between primary and acquired drug resistance among the treated patients. Nevertheless, these reports showed that transmission plays a major role in the epidemic of drug-resistant tuberculosis worldwide. Therefore, emphasis should be placed on developing a prompt case detection to disrupt transmission and thereby blocking the sources of drug resistant tuberculosis.

Our results also showed 40.7% acquired drug resistance; which is presumably caused by therapy failure resulting from several anthropogenic factors, such as poor-quality anti-TB drugs, poor treatment adherence and inadequate or improper treatment^23, 24^. This implies that acquired drug resistance is still a significant factor to be considered in resistance among treated patients. As a preventative measure, we suggest that more efforts and strategies aimed at tackling factors leading to therapy failure should be implemented; more emphasis on the standard management and effective treatment of patients should be placed.

We also observed 56.8% reinfection rate among the 185 patients whose paired isolates were drug sensitive and 28.2% among those that paired isolates were drug resistant. The importance of exogenous re-infection in the propagation of tuberculosis has earlier been highlighted^12, 19, 25^. Based on animal models and the epidemiology of TB in India, Balasubramanian *et al*.^25^ emphasized that reinfection is an essential alternate pathway for TB propagation in endemic areas. Therefore, since there is relatively high rates of exogenous reinfection (both in drug resistant and drug susceptible tuberculosis) as indicated in this study, it could be suggested that physicians may not base diagnosis and treatment on the previous history of tuberculosis of patients but rather on the result of DST. Conversely, the 10.3% (4/39) identical genotype (Fig. 2), observed in the R-S group could be explained on the emerging information that complex sub-clones of *M. tuberculosis* coexist within patients due to microevolution; which may contribute to different responses to drugs by strain from different section of the lungs of the same patient^26^. Mixed infection observed in 7.4% patients with increasing drug resistance may also be a contributing factor in drug resistance among treated patients, which has been shown a greater chance of failing therapy or spreading their undetected strain^27–29^.

We defined acquire drug resistant if they shared the same VNTR genotype. However, because of the lower discriminatory power of VNTR, compared to WGS, several studies show that even with the same VNTR genotype, strains can differ with hundreds of SNPs^30, 31^. In the other hand, Supply^32^ showed that in an outbreak, four out of 94 strains, compared to the core genotype, have a single VNTR locus variance, reminding us that one locus variation to define different genotype could be too strict. In the S-R group, four out of 47 pairs with different VNTR genotypes were differentiated with only one repeat. Because VNTR loci in *M. tuberculosis* more likely evolve via a single repeat^33^, these four pairs possibly indicate the same strains. Nevertheless, these four pairs had minor influence on our main conclusion.

This study has some limitations. We could not completely rule out the possibility that patients be re-infected by the same source though it could be of little chance. And patients’ clinical information, individual patients regimen and epidemiological characteristics of the patients were not available for most of the patients. The 7.8% mixed infection might be under estimated due to low sensitivity of the method.

In conclusion, we have demonstrated a relatively high rate of transmission of drug resistant tuberculosis among treated TB patients in Shanghai, China. Our results implied that transmission of drug resistant strains of *M. tuberculosis* is to great extent prevalent; therefore, strategies aimed at preventing and interrupting transmission are urgently needed to effectively reduce the epidemic of drug resistant tuberculosis.

## Materials and Methods

### Study population

The study population comprised of all TB patients diagnosed and treated in 31 designated district tuberculosis hospitals in Shanghai China during 2009 to 2015. The tuberculosis reference laboratory of the Shanghai Municipal Center of Disease Control and Prevention (SCDC) routinely perform phenotypic DST against isoniazid, rifampicin, ethambutol, and streptomycin of sputum positive culture of *M. tuberculosis* isolates from TB patients treated in these hospitals using the proportion method on Lowenstein-Jensen medium^34^. The *M. tuberculosis* isolates are usually stored in the SCDC culture archives and the phenotypic DST results kept in the center’s database. For this present study, a database search was performed to enroll patients who had at least two available clinical isolates collected longer than 90 days in the Center’s storage facility, and the patients were labeled with study ID.

### Laboratory Procedures

Paired isolates from the same patient were selected and extracted bacterial genomic DNA by the boiled lysis method^35^. Molecular DST determines mutations conferring resistance to rifampin (*rpoB*), isoniazid (*katG, inhA, ahpC*), and second line drugs (*rrs, gyrA, eis* promoter) by multiplex real-time PCR melting curve assay^36, 37^. The VNTR (9 + 3) genotyping was carried out according to the method of Luo et al.^38^. Paired isolates with identical VNTR patterns were further confirmed if whole genome sequencing showed ≤ 12 SNPs between paired isolates^17^. The genomic DNAs for sequencing were extracted following the cetyltrimethyl-ammonium bromide-lysozyme (CTAB) method^39^; a 300-base-pair (bp) paired-end library was constructed for each purified DNA sample; sequencing was performed by Yikon Genomics Co. (Jiangsu, China) and SNPs were called from sequencing data using an in-house Perl script. All methods were carried out in accordance with relevant guidelines and regulations. The sequencing data were deposited in the European Nucleotide  Archive (Accession No. PRJEB21577).

### Definitions

#### Primary Drug Resistance

A patient is considered to have primary drug resistance tuberculosis, when two serial isolates from the patient showed discordant drug susceptibility pattern and different VNTR pattern.

#### Acquired Drug Resistance

This is a situation whereby two serial isolates from one patient had identical genotypes but the first isolate is drug sensitive while the second isolate is drug resistant.

#### Mixed Infection

This is infection with multiple strains of *M. tuberculosis*, distinguishable by their different genotypes, within the same patient before, during or after successful treatment. In this study, the presence of multiple alleles (multiple DNA band) at one or more VNTR locus was taken to indicate mixed infection.

### Ethics

Phenotypic DST was performed as routine work of SCDC, while molecular DST, genotyping and sequencing were done with mask of patients’ name. Patients’ demographic information and testing data were analyzed anonymously and so the study was exempt from ethical review.
